# Capgras Delusion in Late Onset Postpartum Psychosis and Persistent Delusional Disorder

**DOI:** 10.1192/bjo.2024.679

**Published:** 2024-08-01

**Authors:** Derya Nurlu, Rina Gupta, Emily Charlton

**Affiliations:** Essex Partnership University NHS Trust, Chelmsford, United Kingdom

## Abstract

**Aims:**

The Capgras syndrome is one of the four disorders defined under Delusional Misidentification. In the Capgras syndrome the patient believes that someone close to them has been replaced by an imposter pretending to be that person; the abnormality is delusional and not hallucinatory. It is a specific delusion of a person with whom the subject has close emotional ties and towards whom there is a feeling of ambivalence at the time of the onset.

**Methods:**

37 year old Caucasian female presented to the local emergency department 4 months after delivery of her baby. She presented with a suicidal attempt in which she cut her neck and drank bleach. She was convinced that her parents and daughters were replaced by a network and her ex-partner was part of this network. She also believed that the network was out to harm her. She showed other psychotic symptoms along with low mood and hopelessness. Despite being offered high doses of antidepressants and antipsychotics she did not show any improvement hence she was given 12 sessions of ECT. Though this treatment was seen to bring in some benefits, her beliefs were still observed to persist. As her delusions were resistant to treatment and lasted more than 3 months, she was diagnosed to have a Persistent Delusional Disorder.

**Results:**

A literature search showed that Capgras delusions rarely occur in postpartum psychosis. It generally poses a risk to baby's care and wellbeing since in most cases mother either refuses to care for baby or attempts to harm them. Interestingly in this case, mother met her daughter's physical needs but struggled with baby's emotional needs most of the time and was rarely observed to smile and play with her.

**Conclusion:**

In this case report, we present the occurrence of different psychopathologies during postpartum psychosis including Capgras delusion. We underline that this case is different from other cases reported in the literature due to unusual nature of the bond between the mother and baby and the onset of the symptoms.

